# A 3-Year Randomized Trial of Lifestyle Intervention for Cardiovascular Risk Reduction in the Primary Care Setting: The Swedish Björknäs Study

**DOI:** 10.1371/journal.pone.0005195

**Published:** 2009-04-14

**Authors:** Margareta K. Eriksson, Paul W. Franks, Mats Eliasson

**Affiliations:** 1 Björknäs Primary Health Care Centre, Boden, Sweden; 2 Department of Community Medicine and Rehabilitation, Umeå University, Umeå, Sweden; 3 Department of Public Health and Clinical Medicine, Umeå University, Umeå, Sweden; 4 Department of Medicine, Sunderby Hospital, Luleå, Sweden; Leiden University Medical Center, Netherlands

## Abstract

**Background:**

Successfully transferring the findings of expensive and tightly controlled programmes of intensive lifestyle modification to the primary care setting is necessary if such knowledge is to be of clinical utility. The objective of this study was to test whether intensive lifestyle modification, shown previously in tightly-controlled clinical trials to be efficacious for diabetes risk-reduction among high-risk individuals, can reduce cardiovascular risk factor levels in the primary care setting.

**Methodology / Principal Findings:**

The Swedish Björknäs study was a randomized controlled trial conducted from 2003 to 2006 with follow-up on cardiovascular risk factors at 3, 12, 24 and 36 months. A total of 151 middle-aged men and women at moderate- to high-risk of cardiovascular disease from northern Sweden were randomly assigned to either an intensive lifestyle intervention (n = 75) or control (n = 76) group. The intervention was based broadly on the protocol of the Diabetes Prevention Program. The three-month intervention period was administered in the primary care setting and consisted of supervised exercise sessions and diet counselling, followed by regular group meetings during three years. The control group was given general advice about diet and exercise and received standard clinical care. Outcomes were changes in anthropometrics, aerobic fitness, self-reported physical activity, blood pressure, and metabolic traits. At 36 months post-randomisation, intensive lifestyle modification reduced waist circumference (−2.2 cm: p = 0.001), waist-hip ratio (−0.02: p<0.0001), systolic blood pressure (−4.9 mmHg: p = 0.036), and diastolic blood pressure (−1.6 mmHg: p = 0.005), and improved aerobic fitness (5%; p = 0.038). Changes in lipid or glucose values did not differ statistically between groups. At 36 months, self-reported time spent exercising and total physical activity had increased more in the intervention group than in the control group (p<0.001).

**Conclusion / Significance:**

A program of intensive lifestyle modification undertaken in the primary health care setting can favourably influence cardiovascular risk-factor profiles in high-risk individuals.

**Trial Registration:**

ClinicalTrials.gov NCT00486941

## Introduction

Evidence from epidemiological and experimental studies overwhelmingly illustrates the beneficial impact of healthy lifestyle behaviours on cardiovascular risk [1]–[2]. Lifestyle factors such as physical inactivity, diets rich in saturated fats and sugar, and central obesity contribute to the development of type 2 diabetes and cardiovascular disease (CVD) [1]–[3]. People who maintain physically active lifestyles have lower levels of most atherosclerotic and metabolic risk factors [1]–[3], spend more years free from type 2 diabetes [1], and live longer [1]–[2]. A low level of cardiorespiratory fitness, which is influenced by physical activity levels, is a powerful, independent risk factor for premature mortality [1]–[2]. Regular physical activity is also associated with a reduced risk of CVD and with reduced rates of cardiovascular and total mortality in people with type 2 diabetes [1].

A variety of different intervention methods have been used to increase physical activity levels. These include paradigms involving individual- or group-level interventions. The methods to promote physical activity have involved activity feedback using pedometers, activity counseling, and exercise prescription [4]–[6], or more extensive lifestyle programs such as those implemented in the Finnish Diabetes Prevention Study (DPS) [7] and the Diabetes Prevention Program (DPP) [8]. These more extensive lifestyle intervention programs have been shown to induce around 5–7% weight loss and consequently achieve clinically relevant reductions in cardiovascular risk factor levels [9]–[10] and delay the onset of type 2 diabetes [7], [11]–[12] and CVD [1]–[3]. Exercise interventions can reduce central obesity in a dose-dependent manner independently of changes in nutrient or caloric intake [13]–[14], thus improving CVD risk profiles [14]–[15]. In people with type 2 diabetes, both structured exercise training [16]–[19] and physical activity counseling [20]–[21] result in weight loss and improved metabolic homeostasis. Although weight loss certainly mediates many of the beneficial effects of exercise on type 2 diabetes risk reduction, exercise may also prevent or delay the onset of type 2 diabetes through independent mechanisms [22] involving insulin signaling, glucose and lipid oxidation, and reduced hepatic glucose production.

Although a number of randomized controlled trials have successfully demonstrated the efficacy of intensive lifestyle intervention for cardiovascular [23]–[25] and type 2 diabetes [11]–[12] risk reduction, to our knowledge only a few attempts to apply the protocols used in these studies for lifestyle modification in the primary health-care setting have been documented [26]–[27], none of which were randomized or involved long-term follow-up.

We recently reported the provisional results (at 12 months follow-up) of a randomized controlled trial, where individuals at moderate- to high-risk of cardiovascular disease were randomly assigned to receive either intensive lifestyle modification or standard care [28]. In that study, which was run from a primary health-care centre in northern Sweden, we were able to demonstrate that lifestyle modification improves cardiovascular risk factor levels. The intervention was group-based, focusing on aerobic and resistance training, improvements in dietary habits, motivation, and maintenance of behavioral changes, and was carried out without additional resources at a primary-care centre. In this report from the same clinical trial, we describe the overall trial results from baseline through three years follow-up.

## Methods

The protocol for this trial and supporting CONSORT checklist are available as supporting information; see Checklist S1 and Protocol S1.

**Figure 1:**
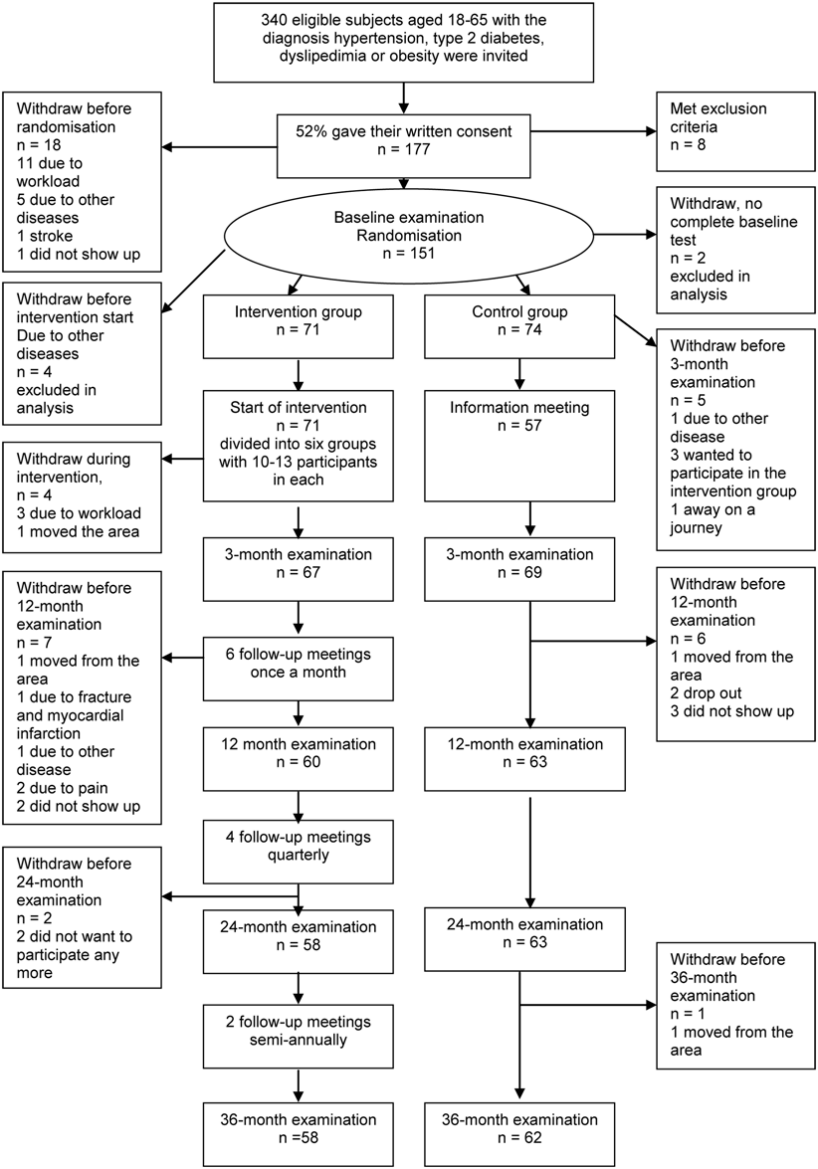
Participants flow Diagram.

The Swedish Björknäs Study was a randomized controlled clinical trial. Participants assigned to the control arm of the study received standard care and those assigned to the intervention arm received a program of lifestyle modification including structured exercise training sessions and diet counselling. Individuals were followed-up at 3, 12, 24 and 36 months (Figure 1.)

### Participants

The study population was selected from the catchment area of the Swedish Björknäs primary health care center in the town of Boden in northern Sweden. Participants were enrolled in the study by the medical supervisor of the health care at the center. All individuals aged 18–65 years with a clinically documented diagnosis of hypertension, dyslipidemia, type 2 diabetes, obesity or any combinations thereof were identified from computerised case records. Individuals with a diagnosis of coronary heart disease, stroke, transient ischemic attack, severe hypertension (SBP>180 or DBP>105 mmHg), dementia or severe psychiatric morbidity were excluded. The remaining 340 eligible individuals were invited by letter to participate in the trial (Figure 1). Of those, 177 individuals agreed to participate (52% of those eligible); 18 withdrew before randomisation, and a further eight met the study's exclusion criteria.

### Randomization

A total of 151 enrolled participants were randomly allocated using a computer-generated random numbers sequence to the intervention group (n = 75) or the control group (n = 76). An independent statistician generated the allocation sequence and randomisation numbers were kept in sealed, opaque envelopes. The allocation was concealed until after the baseline examinations were completed and participants were assigned to their groups by the research physiotherapist. After excluding four subjects who were allocated to the lifestyle intervention but did not start the intervention period, and excluding two subjects with incomplete baseline data allocated to control group, the numbers of participants were 71 and 74 in the intervention and control groups, respectively.

### Ethics

All participants provided written informed consent to participate and the study protocol was approved by institutional review board of Umeå University, Sweden.

### Clinical examination

At each examination, measurements of weight, height, circumferences of the waist and hips, blood pressure by a standard auscultatory method, and an estimate of maximal oxygen uptake (VO_2_max) were obtained. Body mass index (BMI: weight in kg / height in meters squared) and waist-to-hip ratio were calculated. VO_2_max was estimated as described by Åstrand [29] from each participant's individual heart rate response to a given submaximal workload (i.e. 50–150 W, depending on the participant's weight and self-reported physical activity) using a bicycle ergometer (Monark, Varberg, Sweden) and recorded at steady-state with a heart rate of ≥120 beats/min. In the original test Åstrand reported a correlation of r = 0.78 between estimated and measured VO_2_max [29]. Other authors have reported correlations within the range r = 0.69–0.95, varying by age and gender. The method has also proven reliable when repeated measures are available within the same individual [30].

A history and physical exam was undertaken at baseline focusing on cardiovascular disease and current medication usage. Changes in pharmacological treatment were followed yearly. A trained research physiotherapist (ME) performed all interviews and undertook the anthropometric and blood pressure measurements to minimize observer bias. Two other trained physiotherapists performed the exercise stress tests. The physiotherapists were not blinded to allocation of treatment at the follow-up examinations.

### Laboratory measurements

The methods for blood collection, storage and analysis have been described in detail previously [28]. In brief, total cholesterol and triglycerides were analysed by enzymatic colorimetry (slide method, Vitros 5.1.Ortho-Clinical diagnostics, Raritan, New Jersey). High-density lipoprotein cholesterol (HDL-C) was analysed by enzymatic (dextran sulfate procedure) colorimetry (Hitachi 917, Roche Diagnostics Scandinavia AB, Bromma, Sweden). Low-density lipoprotein (LDL) cholesterol was calculated using the Friedwald equation. Serum glucose was analysed by enzymatic (glucose oxidase) colorimetry (Vitros 5.1, Ortho-Clinical Diagnostics, Raritan, New Jersey). Glycosylated haemoglobin (Hb) A1c was analysed by High Performance Liquid Chromatography, Ion exchange Chromatography, Photometry (VARIANT™II, BIO-RAD laboratories, Hercules, California). All biochemical analyses were performed at the clinical chemistry laboratory at Sunderby Hospital, Luleå, Sweden. All non-diabetic participants underwent a 75 g oral glucose tolerance test, using the protocol recommended by the WHO, at two and three years post-randomisation.

### Behaviour assessments

Physical activity and tobacco habits were assessed using a self-administered questionnaire, previously used in the national project “physical activity on prescription” by the Swedish Institute of Public Health [31]. The questionnaire has also been used by a number of Swedish regional health authorities to assess public health within the community. However, no validation studies have been performed for this questionnaire. The questionnaire is intended to characterise a participant's levels of leisure time activity (LTPA), structured exercise, and total physical activity (TPA). LTPA includes activities such as housekeeping, gardening, walking or biking to work, and ‘exercise’ includes structured activities such as aerobics, jogging, swimming, and ball games carried out during a normal week. Four activity levels were determined for LTPA and exercise; ‘none’, ‘<30 min/d’, ‘30–60 min/d’ and ‘>60 min/d’. Nine questions on TPA were included that were intended to estimate all forms of physical activity undertaken during the past 12 months. A TPA index with four activity levels was constructed: ‘sedentary’, ‘minimally active’, ‘moderately active’, and ‘very active’. Participants in both groups were also requested to complete physical activity logs.

### Interventions

The lifestyle intervention consisted of supervised exercise training and diet counselling, followed by regular group meetings. The first three months of the intervention included three sessions per week of supervised progressive exercise training and diet counselling on a total of five occasions. The exercise training sessions consisted of aerobic exercises such as Nordic walking (brisk walking with poles), interval training on a bicycle ergometer, circuit-type resistance exercise, and aqua-aerobics. These activities were led by physiotherapists and physiotherapy assistants at the primary care centre. The physiotherapists were responsible for both the clinical examinations and delivering the exercise intervention. Exercise training was performed in small groups (n = 10–13) with each group comprising participants of similar ages and fitness levels. All groups were offered one session of each activity every week. The exercise training sessions lasted 40–45 min during the first month and increased to 60 min during the second and the third months. The duration of the bicycle ergometry exercise was increased from 20 min to 30 min after one month. The resistance training consisted of 12 different movements per circuit: two sets of 10–15 repetitions were performed at each station. The load was individualized and increased over time as the participant's strength improved. All programmes included a warm-up and a cool-down period with stretching. The aim of the exercise sessions was to increase cardiorespiratory fitness and to improve functional capacity and strength of the large muscle groups of the arms, torso and legs. To approximate optimal exercise intensity, the Borg scale of perceived exertion was used [32]. A moderate intensity (60–80% of maximal heart rate) corresponding to 13–15 on the Borg scale was recommended for all activities.

A trained dietician was responsible for the diet counselling. The dietary advice was given in small groups but no individual counselling was provided. The participants received both written and verbal dietary information. The counselling was in accordance with the Nordic nutrition recommendations [33]. Briefly the participants were encouraged to increase their intake of fish, fruits, vegetables fibre-rich products and complex carbohydrates. Participants were also advised to restrict total caloric intake by reducing consumption of sugar and saturated fat and to use low-fat milk products, soft margarines and vegetable oils rich in monosaturated fatty acids (See table 1).

**Table 1:** Summary of physical activity and diet recommendations provided to participants randomized to the control and intervention groups.

	Control group (standard recommendations)	Intervention group (extended recommendations)
**Physical activity**	- Aim to accumulate at least 30 minutes of moderate-intensity physical activity on most, preferably all, days of the week	- Aim to accumulate at least 30 minutes of moderate-intensity physical activity on most, preferably all, days of the week
Swedish Institute of Public Health	- Additional health benefits can be achieved by extending the time spent in moderate-intensity activities, or by increasing the intensity of actives	- Additional health benefits can be achieved by extending the time spent in moderate-intensity activities, or by increasing the intensity of actives
		- Aim to undertake 20–30 minutes of moderate to vigorous intensity of aerobic activities lasting 20–30 minutes, three times each week (e.g. bicycle ergometry exercise, Nordic walking, aqua-aerobics)
		- Aim to undertake muscle-strengthening activities, lasting 20–30 minutes, at least twice each week (e.g. circuit-type resistance exercise, aqua-aerobics)
**Diet**	Energy percent, E%,	Energy percent, E%,
	- Carbohydrates, 55% (50–60)	- Carbohydrates, 55% (50–60)
National food administration	sugar <10%	sugar <10%
	- Protein, 15% (10–20)	- Protein, 15% (10–20)
(NNR)	- Fat, 30% (25–35)	- Fat, 30% (25–35)
	saturated fat and trans fat, 10%	saturated fat and trans fat, 10%
	monounsaturated fat, 10–15%	monounsaturated fat, 10–15%
	polyunsaturated fat, 5 (−10)	polyunsaturated fat, 5 (−10)
	General advice:	General advice:
	- Increase intake of fish, fruits, vegetables, fibre rich products and complex carbohydrates.	- Increase intake of fish, fruits and vegetables, fibre rich products and complex carbohydrates.
	- Reduce consumption of sugar and saturated fat.	- Reduce consumption of sugar and saturated fat.
	- Use low-fat milk products, soft margarines and vegetable oils rich in monosaturated fatty acids.	- Use low-fat milk products, soft margarines and vegetable oils rich in monosaturated fatty acids.
	- Restrict total caloric intake.	- Restrict total caloric intake.
	- Regular meal distribution.	- Regular meal distribution.
		Information about meal content and function of:
		- Fat, carbohydrates, protein, vitamins, minerals and antioxidants.
		- Energy balance and energy expenditure.
		- Alcohol.
		Cooking advise

After the active intervention period, participants from each training group were invited to attend regular follow-up meetings on six occasions during the first year (from September to February), on four occasions during the second year and on two occasions during the third year (Figure 1). The objectives of the follow-up meetings were (i) to improve the participant's knowledge about the relationships between lifestyle and health, (ii) to encourage participants to favourably modify their lifestyles, and (iii) to provide social support and to facilitate adherence to the intervention. The stages-of-change model of behavioural change was used as theoretical basis for the intervention [34]. The techniques used were standardized for all participants. After the active intervention period most individuals were in the ‘preparation’ or ‘action’ phases. Participants were asked to investigate benefits, barriers, and costs of adherence to a healthier lifestyle and were encouraged to establish individual goals for weight reduction and to develop a personal physical activity plan. They were encouraged to maintain at least 30 min/day of physical activity. Participants also received information about community-based physical activity and a study visit at a local gym was provided for each training group.

At the group meetings discussions focussed on physical activity, diet, coping with stress, and the effects of tobacco on health. The progress of each participant was discussed at the meetings during the follow-up period. Emphasis was placed on identifying situations that inhibit healthy behaviours such as holidays or heavy workloads and strategies to handle these situations were discussed. During the final stage of the trial, the importance of maintaining healthy habits and on avoiding relapses was reinforced.

Physiotherapists supervised most of the in-person meetings, and a dietician provided further diet counselling on three occasions, once each year during the follow-up period. The medically responsible physician took part in one meeting at the beginning of the study and one at the end of the study to answer participants' questions about the health aspects of the intervention and to reinforce the messages being delivered by the physiotherapists and the dietician.

Participants in the standard care control group were given verbal and written information about healthy behaviours, including exercise and diet. This information was delivered by the physician, a physiotherapist and a dietician at a group meeting following the baseline examination (Figure 1).

### Blinding

Neither the participants nor the family practitioners or physiotherapists who delivered the intervention were blinded to the allocation of treatment. Each year, participants in both groups were given a written summary of their laboratory and clinical test results. Participants in both groups continued with their routine care, delivered for example by their family practitioner or specialist, throughout the study, and no special instructions were given regarding other preventive measures.

### Objectives

Our objective was to test the hypothesis that a lifestyle intervention program in the primary health care setting facilitates long-term (up to 3 yrs) clinically relevant improvements in cardiovascular and metabolic risk profiles in people who, at baseline, were at high risk of cardiovascular disease.

### Outcomes

Outcome measures were changes in anthropometry (BMI, weight, waist, waist-to-hip ratio), VO_2_max, self-reported physical activity, blood pressure, triglycerides, cholesterols (total, HDL, and LDL), fasting blood glucose, glucose tolerance, and HbA1c.

### Sample size

In the DPP, weight loss at 1 and 3.4 years within the lifestyle intervention group averaged 6.8 kg and 4.1 kg, respectively [33]. For sample size calculations, we assumed 20% of participants from our study would withdraw before completion, and that our intervention would yield approximately half the weight loss at three years post-randomisation than observed in the DPP (i.e. 2.1 kg, SD ±1 kg). We also assumed that because the control group was receiving standard health advice and was not blinded to treatment allocation, those in that group would lose up to 1 kg (SD ±1 kg) in weight during the trial. We selected a two-sided p-value of 0.001 to account for multiple statistical comparisons. With these parameters, our study was powered at ∼99% to detect the predicted difference in weight change between groups at 3 years follow-up. Power was calculated using STATA v9.2 SE (StataCorp, TX, USA)

### Statistical methods

SPSS for Windows (v15.0, Chicago, IL) and SAS for Windows (V9.1, Carey, NC) were used for all statistical analyses. Data were analysed on an intention-to treat (ITT) basis, regardless of adherence to the intervention and included all randomly allocated persons, with the exception of the six individuals excluded for the reasons given above (Figure 1). If data were missing, the last observation was carried forward.

Generalized linear models with repeated measures analysis of variance and univariate tests of variance were used to investigate changes in the dependent variables (as continuous traits) over time at each of the follow-up time points, adjusted for baseline values. To determinate the independent effects of the intervention on blood pressures, lipids, or HbA1c, these variables were adjusted for “medication load”. To this end, a variable was computed from the number of specific drugs prescribed to treat the respective trait by assigning values of 1 for low doses and 2 for high doses and subsequently summing these values.

### Ancillary analyses

Mixed-model analyses were used to calculate the adjusted effects of the intervention at each of the follow-up points for continuous trait outcomes and to investigate the overall effect of the intervention. The mixed-model included all available follow-up data. To account for multiple observations within individual, models were fitted using an unstructured covariance matrix. The choice of matrix was determined by comparing Akaike's Information Criterion for different structures and selecting the matrix that best fitted the data. This method allows for trait correlations between different examinations within the same individual. The results of these models indicate whether changes in the values of a trait from baseline to follow-up differ significantly between the intervention and the control groups. In addition to the fixed effects, the mixed-model estimates components of variance (random effects) representing the relation between different observations for the same individual. For continuous or rank-ordered data, the parameters of these variables were estimated by a maximum-likelihood procedure. For ordinal data such as those obtained from questionnaires, the non-parametric Mann-Whitney U test was used for analysis between groups at each follow-up time point, and mixed-models were used to assess the overall effects of the intervention. For other discrete variables, the Pearson Chi-Square test and repeated measures analysis were used. A p-value <0.05 was considerer statistically significant.

## Results

### Recruitment

Participants were recruited in January 2003 and underwent baseline examinations in February 2003. Follow-up examinations were conducted 3, 12, 24 and 36 months after the baseline examination. The study was completed in March 2006.

### Participants flow

From the original 151 volunteers, a total of 120 completed the 3-year follow-up examination (80%); n = 58/75 in the intervention group and n = 60/76 in the control group. Attrition was greatest during the first year of the study, attributed mainly to ill health, heavy workloads, or relocation to another geographic region during follow-up (Figure 1).

### Subject characteristics

There were no differences in demographic characteristics or CVD risk profiles between study participants and the 163 subjects who declined to participate. Mean age in the study group was 54.4 vs. 51.8 years in the group of non-participants. Gender distribution and the prevalence of risk factors were similar. In the study group 43% were male, 66% had a diagnosis of hypertension, 28% had a diagnosis of type 2 diabetes, and 22% had dyslipedemia. The corresponding proportions among those who did not participated were male 50%, hypertension 69%, type 2 diabetes 32%, and dyslipidemia 18%. Of all eligible subjects about half had at least one CVD risk factor and a third had two or more CVD risk factors besides being overweight or obese. Only 40 of the 340 individuals had “overweight” or “obesity” recorded as clinical diagnoses in their case records, which were an underestimate of the true prevalence of these conditions (see Table 2).

**Table 2:** Baseline Characteristics of randomised participants in the Swedish Björknäs Study*.

Characteristic	Intervention group (n = 71)	Control group (n = 74)
**Age**, yr, mean (SD)	55.7 (6.6)	53.1 (8.2)
**Sex**, No. (%)		
Male	35 (49)	27 (36.5)
Female	36 (51)	47 (63.5)
**Non-smokers**, No. (%)	54 (76)	61 (82)
Ex-smokers, No. (%)	24 (34)	29 (39)
**Anthropometrics**, mean (SD)		
Body weight, kg	87.4 (16.5)	84.3 (20.0)
Body Mass Index, Kg/m^2^ †	30.2 (5.2)	29.4 (5.1)
Waist circumference, cm	104.1 (13.2)	100.2 (15.9)
Hip circumference, cm	108.6 (10.2)	107.4 (8.6)
Waist-to-hip ratio	0.96 (0.08)	0.93 (0.09)
**Presence of overweight or obesity**, No. (%)		
Body Mass Index 25–29,9	32 (45)	32 (43)
Body Mass Index ≥30	32 (45)	30 (41)
**Blood pressure**, mean (SD)		
Systolic, mmHg	146 (15.5)	145 (17.6)
Diastolic, mmHg	88 (7.1)	87 (8.4)
**Cardiovascular risk factors**, mean (SD)		
Total Cholesterol, mmol/l	5.49 (1.05)	5.43 (0.91)
High Density Lipoprotein Cholesterol, mmol/L	1.39 (0.32)	1.46 (0.40)
Low Density Lipoprotein Cholesterol, mmol/L	3.17 (0.91)	3.12 (0.82)
Triglycerides, mmol/l	2.08 (1.24)	1.90 (1.15)
Fasting-blood glucose, mmol/L ‡	5.24 (0.50)	5.20 (0.50)
HbA1c (%) §	6.30 (1.35)	6.62 (2.05)
**Type 2 diabetes**, No. (%)	23 (32)	17 (23)
**Exercise test variables**, mean (SD)		
Maximal Oxygen uptake, VO_2_ , L/min ¶	2.1 (0.6)	2.2 (0.5)
Maximal Oxygen uptake, VO_2_ , mL/kg per minute ¶	25.4 (6.4)	25.8 (6.0)
**Total physical activity**, No. (%)		
Sedentary	14 (20)	3 (4)
Minimally active	27 (38)	35 (47)
Moderately active	22 (31)	25 (34)
Very active	8 (11)	11 (15)
**Exercise**, No. (%)		
None	43 (61)	37 (50)
<30 min/d	20 (28)	22 (30)
30–60 min/d	8 (11)	13 (18)
>60 min/d	0 (0)	2 (3)
**Leisure-time activity**, No. (%)		
None	14 (20)	5 (7)
<30 min/d	23 (32)	25 (34)
30–60 min/d	30 (43)	29 (39)
>60 min/d	4 (6)	15 (20)

* Age and anthropometric, clinical and metabolic data are given as mean (SD); for other variables data are given as the number of observations (%).

† Calculated as weight in kilograms divided by the square of height in meters.

‡ n = 49/57.

§ n = 22/17 only in known diabetics.

¶ n = 50/42.

In the intervention group 12 women and 5 men withdrew, compared with 5 women and 9 men in the control group. Mean age at recruitment among individuals who withdrew was 53.2 years, which was not statistically different from those who completed the trial (mean age 55.2 years). In both groups the individuals who withdrew were obese or overweight, with one or two additional CVD risk factors. Those who withdrew before randomisation due to employment commitments were somewhat younger (mean age 49.7 years) and most were female and had irregular working hours. In contrast, subjects who withdraw due to illness were older (mean age 61.8 years).

### Compliance

Seventy percent of the intervention group attended the supervised exercise sessions during the three-month intervention period, but varied from 15% to 100% across sessions. Sixty-four percent took part in 3–5 diet-counselling sessions but 36% participated on only one or two occasions. Low attendance was mainly due to employment commitments. During the first year of the trial mean attendance at the follow-up meetings was 70%, during the second year 63% and at year three 66%. Seventy-seven percent of the control group attended the information meeting about lifestyle and health (Figure 1). Participants in both groups were asked to complete activity logs during the trial. Of the 120 participants who completed the 3-year follow-up examination, 60% of the intervention group completed activity logs during the first year, 40% during the second year and 31% during the third year. In the control group the corresponding proportions were 46%, 38% and 29%.

### Baseline data

Although participants were randomly assigned to the study arms, not all characteristics were distributed equally between the control and intervention groups. As shown in Table 2 and Table 3, at baseline participants in the intervention group tended to have larger waists, undertook less LTPA and were more frequently treated with lipid lowering drugs than those in the control group.

**Table 3:** Changes in medication treatment and cigarette smoking from baseline to 3 years follow-up in the Swedish Björknäs study*.

Characteristic	Intervention group (n = 71) †	Control group (n = 74) †	P-Value‡	P-value §
**Current cigarette smoking**, No. (%)				
Baseline	17 (24)	13 (18)	0.34	
3 month	16 (23)	13 (18)	0.46	
1 year	14 (20)	13 (18)	0.74	
2 years	12 (17)	13 (18)	0.92	
3 years	10 (14)	12 (16)	0.72	0.04
**Hypertension**, medication load 0–7, M (SD)				
Baseline	1.42 (1.58)	1.50 (1.50)	0.45	
3 months	1.44 (1.61)	1.49 (1.53)	0.50	
1 year	1.58 (1.77)	1.62 (1.61)	0.61	
2 years	1.75 (1.80)	1.82 (1.63)	0.30	
3 years	1.90 (1.88)	1.88 (1.69)	0.63	0.65
**Dyslipidemia**, medication load 0–2, M (SD)				
Baseline	0.39 (0.60)	0.15 (0.46)	0.002	
3 months	0.39 (0.60)	0.18 (0.48)	0.011	
1 year	0.46 (0.61)	0.20 (0.52)	0.001	
2 years	0.51 (0.58)	0.23 (0.54)	0.000	
3 years	0.55 (0.58)	0.28 (0.63)	0.000	0.41
**Diabetes mellitus and Impaired glucose tolerance at Oral glucose tolerance test**, No.(%)				
2 years ¶	9 (24)	15 (31)	0.52	
3 years ¥	9 (24)	16 (33)	0.37	

* Data are given as the number of observations (%) or mean (SD).

† Last observation carried forward for individuals who withdrew. Number of participants at 3 months: IG = 66, CG = 69. n at 1 year IG = 60, CG = 63. Number of participants at 2 years: IG = 58, CG = 63. n at 3 year IG = 58, CG = 62.

‡ P- value for difference between groups generated using the Pearson Chi-Square test.

§ P- value generated using a general linear model with repeated measures.

¶ IG/CG n = 37/49.

¥ IG/CG n = 37/48.

### Numbers analysed

All data analyses were performed on an ITT basis. Accordingly, if data were missing, the last observation was carried forward. Analyses included 71 participants from the intervention group and 74 participants from the control group, except in analyses of fasting blood glucose, HbA1c and VO_2_max (Table 2). Fasting blood glucose concentrations were analysed only in participants without known diabetes and HbA1c was analysed in participants with diagnosed diabetes. Participants on beta-blockers were excluded from analyses where VO_2_max was the outcome, as the heart rate response to exercise (a necessary component of the VO_2_max prediction equation) is blunted with this type of medication.

### Outcomes and estimation

**Figure 2:**
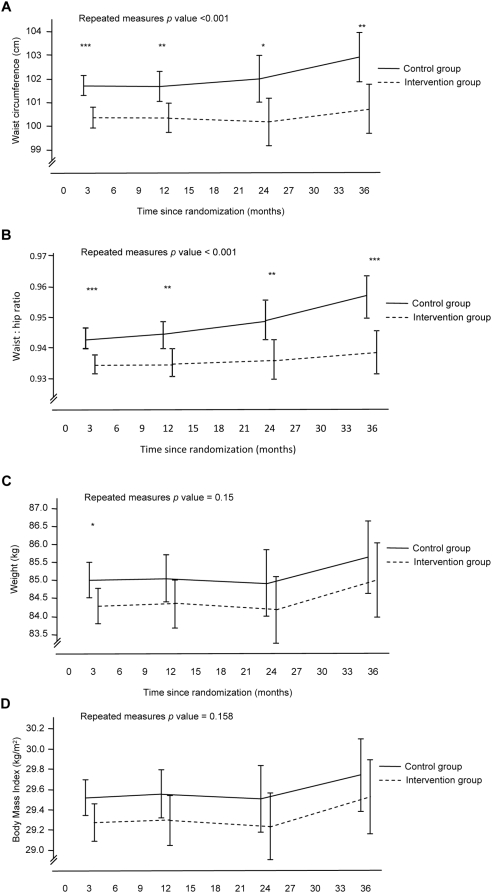
(A–D) Changes in anthropometrics. Data are adjusted means (95% confidence intervals) for each time point derived from generalised line ar models with repeated measures. Adjustments are made for the baseline value for the respective outcome variable. Blood pressure values are also adjusted for medication load. Statistical significance at each follow-up time point generated from univariate analysis of variance, * p<0.05, ** p<0.01, *** p<0.001.

At baseline, the proportions of normal weight, overweight and obese individuals in the intervention group were 10%, 45% and 45%, respectively; in the control group, the corresponding proportions were 16%, 43% and 41% (Table 2). Improvements in anthropometric characteristics were evident in both groups at three years post-randomisation, although improvements were generally greater in the intervention group (Figure 2). Statistically significant reductions in weight between groups were observed at the 3-month follow-up exam. The proportion of participants classified as normal weight (i.e., BMI 20–24.9 kg/m^2^) in the intervention group increased from 10% (n = 7) to 17% (n = 12) during the 36 months of the trial, although this change did not differ significantly from the control group.

The reduction in waist circumference (ITT p = 0.001; mixed model p<0.0001) and waist-to-hip ratio (ITT p<0.0001; mixed model p<0.0001) was greater in the intervention group than in the control group. Values in the intervention group were lower at each follow-up point (Figure 2). At 36 months post-randomisation, waist circumference (−2.2 cm) and waist-to hip ratio (−0.02) had decreased in the lifestyle intervention group. In the control group waist circumference was 102.9 cm (95%CI 101.87–103.92) at 3-year, and in the intervention group 100.7 cm (95% CI 99.65–101.74). No change was noted in hip circumference (p = 0.178) (mixed-model p-value 0.36).

**Figure 3:**
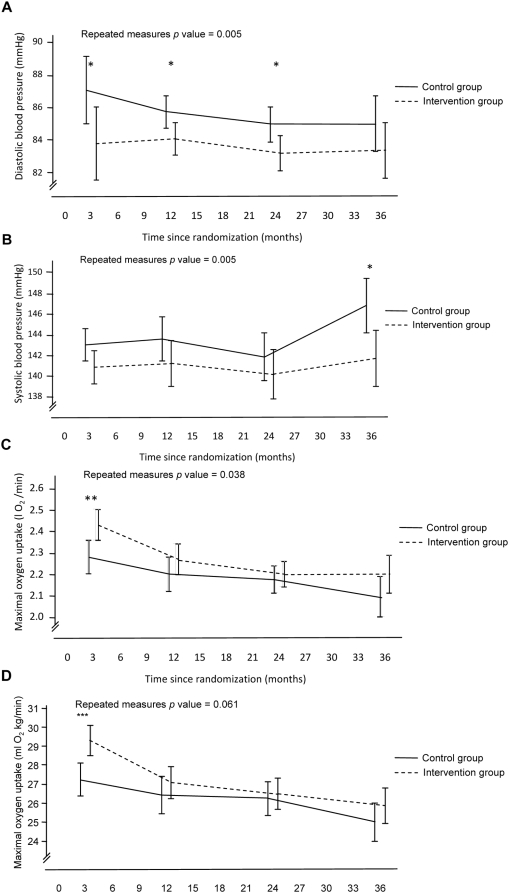
(A–D) Changes in clinical characteristics. Data are adjusted means (95% confidence intervals) for each time point derived from generalised linear models with repeated measures. Adjustments are made for the baseline value for the respective outcome variable. Blood pressure values are also adjusted for medication load. Statistical significance at each follow-up time point generated from univariate analysis of variance, * p<0.05, ** p<0.01, *** p<0.001.

Reductions in systolic (ITT p = 0.03; mixed model p = 0.0062) and diastolic (ITT p = 0.005; mixed model p = 0.0004) blood pressures were greater in the intervention group than in the control group, after adjustments for baseline values and medication load. Systolic blood pressure was significantly lower (−4.9 mmHg) in the intervention compared with the control group after three years (mean 141.7 mmHg; 95% CI 139.0–144.4 mmHg vs. mean 146.8 mmHg; 95%CI 144.2–149.4 mmHg). Diastolic blood pressure was significantly lower in the intervention group at all time points except at three years (Figure 3). During follow-up, the use of blood pressure lowering drugs increased in both groups; this increase did not differ between groups (Table 3).

**Figure 4:**
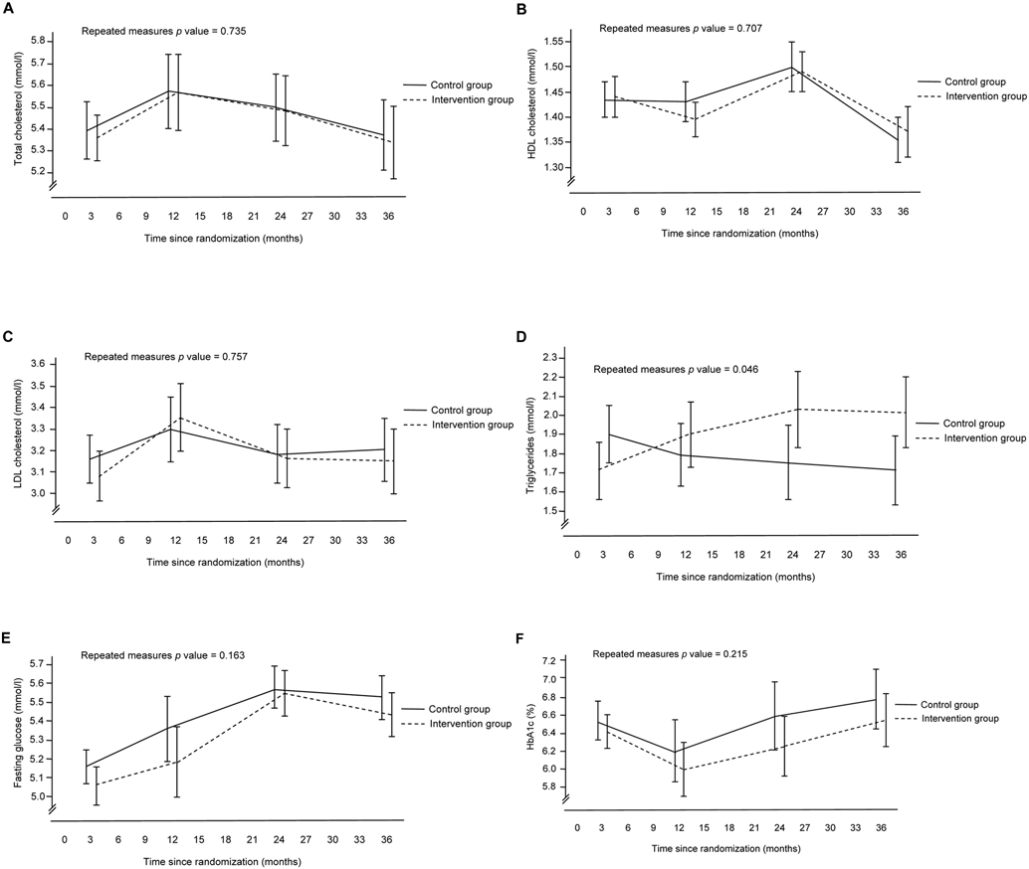
(A–F) Changes in laboratory characteristics. Data are adjusted means (95% confidence intervals) for each time point derived from generalised linear models with repeated measures. Adjustments are made for the baseline value for the respective outcome variable. Blood pressure values are also adjusted for medication load. Statistical significance at each follow-up time point generated from univariate analysis of variance, * p<0.05, ** p<0.01, *** p<0.001.

No significant differences in lipid variables were observed between groups during follow-up (Figure 4). The medication load of lipid lowering drugs was higher in the intervention group than in the control group at all time points (Table 3).

In participants with known diabetes, no differences between groups were observed in HbA1c levels or medication usage during follow-up and no differences between groups were observed in fasting-blood glucose levels in participants without known diabetes (Figure 4). However, one new clinical diagnosis of diabetes occurred in the control group and none was observed in the intervention group. An OGTT was carried out at the examinations after 2 and 3 years. The prevalence of impaired glucose tolerance and diabetes diagnosed by OGTT tended to be lower in the intervention group (24%) than in the control group (34%), although these proportions were not statistically different (Table 3).

After an initial improvement in estimated maximal oxygen uptake in both groups, which was greatest in the intervention group (VO_2_max 0.3 l/min; p = 0.006, 4.0 ml/kg/min; p<0.001), a gradual decline toward baseline values was observed during follow-up (Figure 3). In the ITT analysis, the improvement by study end in estimated maximal oxygen uptake was greater in the intervention group (VO_2_max 2.2 l/min; 95% CI 2.11–2.29) than in the control group (VO_2_max 2.1 l/min; 95% CI 2.00–2.19) when expressed in absolute terms (p = 0.038), although not when expressed relative to body mass (VO_2_max 26.0 ml/kg/min; 95%CI 25.0–26.9 v s 25.0 ml/kg/min; 95% CI 24.0–26.1) (p = 0.061). In the mixed model analysis, the differences in VO_2_max reflected those observed in the ITT analyses (VO_2_max l/min: p = 0.049; VO_2_max ml/kg/min: p = 0.12).

**Figure 5:**
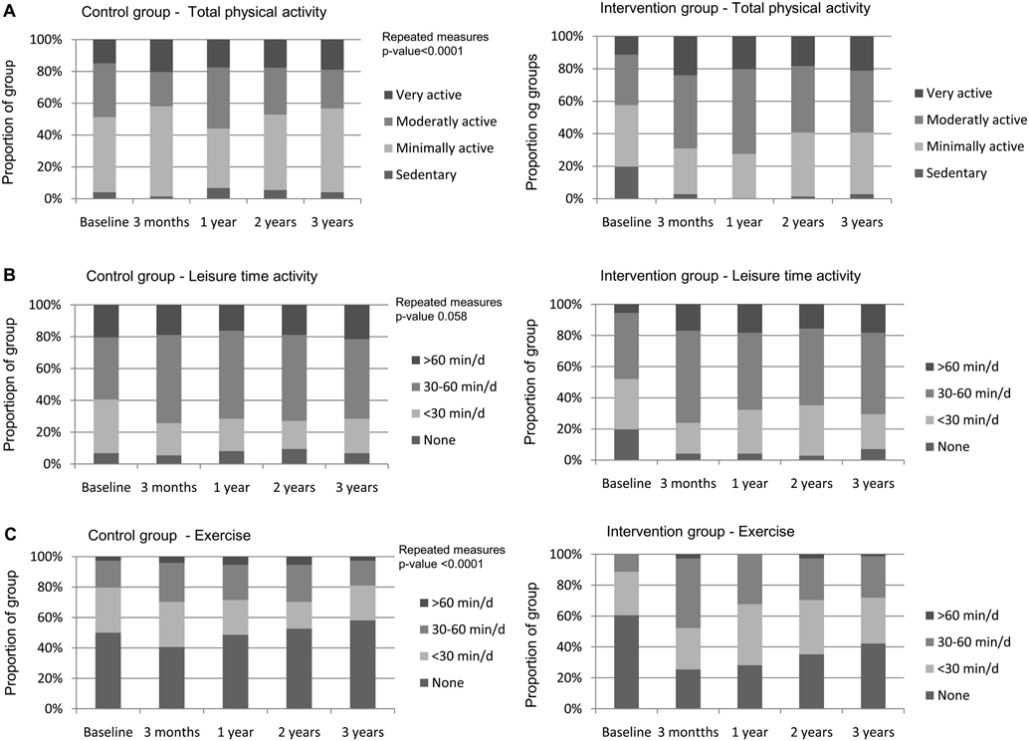
(A–C) Changes in physical activity level. Proportion of participants reporting the level of each variable, total physical activity, leisure time physical activity and exercise and ordered as follows: TPA; sedentary, minimally active, moderately active and very active. LTPA and exercise; ‘none’ = 0, ‘<30 min/day’ = 1, ‘30–60 min/day’ = 2, and ‘>60 min/day’ = 3. P values from general linear model repeated measures analysis.

The improvements in VO_2_max may reflect an effect of increased physical activity levels in participants in the intervention group; those individuals reported more exercise participation (p<0.0001) and greater TPA (p<0.0001) compared with participants in the control group by the end of the trial. At baseline 20% of the lifestyle intervention group was sedentary, whereas only 7% was sedentary at the 3-year examination. At three years about 70% of both groups reported LTPA of at least 30 min/d, but in the intervention group the proportions of those reporting being moderately or very physical active had increased from 42% to 59%, respectively. This contrasted the control group, where physical activity had decreased from 49% to 43%, respectively. Correspondingly, in the intervention group the proportion reporting exercising at least 30 min/d or more increased from 11% to 28% (Figure 5). The results from the mixed-model analyses were consistent with the ITT analyses for both exercise (p<0.001) and TPA (p = 0.0009).

The proportion of individuals who quit smoking in the intervention group (41%) was significantly greater than in the control group (8%) (p = 0.04) (Table 3).

### Adverse advents

No adverse events (i.e. fractures, sprains, or serious cardiovascular events associated with exercise) were reported by participants during the study.

## Discussion

We present here the full results from the Swedish Björknäs Study, a three year randomised controlled trial of intensive lifestyle modification for cardiovascular risk reduction in patients at moderate- to high-risk of cardiovascular disease. The study protocol was adapted from the DPP, but was delivered with limited resources at a primary health care centre in the northern Swedish town of Boden. The lifestyle intervention favourably impacted physical activity and aerobic fitness, waist circumference, waist-to hip ratio, blood pressure, and smoking cessation. No effect of the intervention was observed on glycaemia or lipidaemia.

Being obese or having diabetes diminishes quality of life [35]–[36] and imposes considerable economic burdens on health care systems [35]. Thus, interventions that beneficially impact cardiovascular risk-factor levels in high-risk individuals are likely to improve individual well-being, may be more cost-effective than population-wide lifestyle modification strategies [37], and safer and less expensive than drug therapy [38]. Several large-scale intervention studies during the past decade have illustrated the efficacy of lifestyle intervention for diabetes risk reduction. In the DPP [8] and the Finnish DPS [7], the reduction in diabetes risk attributable to lifestyle modification was 58% in both studies using ITT analyses. The DPP authors predicted that in individuals with similar characteristics to the DPP volunteers, seven would need to participate in a similar lifestyle intervention program to prevent one case of diabetes during a 3-year follow-up period [8]. In the DPP, the level of diabetes risk reduction in people who met the goals for weight loss, physical activity and diet modification was 89% [39]. Other reports from the DPP have highlighted the beneficial impact of lifestyle modification on other cardiovascular risk factors including glucose homeostasis, insulin sensitivity, lipid levels, inflammation, coagulation, and obesity [40]. These observations illustrate the remarkable potential of lifestyle change for diabetes and cardiovascular risk reduction.

Notwithstanding the considerable health benefits conferred by the DPP lifestyle intervention, the protocol was costly and, as delivered in the DPP, beyond the scope of many primary health care agencies (for further details, see: www.bsc.gwu.edu/dpp/lifestyle/dpp_part.html). The within-trial costs of the DPP lifestyle intervention totalled $24400 (USD) per case of diabetes prevented or delayed [41]. By the conclusion of the initial intervention period, the running costs of the DPP had totalled $174.3 million (USD) (http://www.nlm.nih.gov/databases/alerts/diabetes01.html). An important and hitherto largely unanswered question is whether the DPP protocol of lifestyle intervention is effective outside a tightly controlled and well-funded setting of an extensively supported multi-centre randomised clinical trial. Although a small number of studies have attempted to address this question [42]–[43], all were non-randomised and none was carried out in the primary care setting with long-term follow-up.

### Interpretation

In an earlier publication from the Swedish Björknäs Study we reported the interim (one year) results of the trial [28]. We presented crude between-group comparisons showing favourable effects on waist circumference, waist-hip-ratio and diastolic blood pressure. In the present report, we show that through reductions in central obesity, physical activity and smoking cessation, the lifestyle intervention also favourably impacted blood pressure levels. However, we did not observe improvements in glycemic control or weight reduction, which contrasts the results from the DPP and Finnish DPS. The lack of impact on glucose homeostasis in the present study may be explained by the relatively modest level of weight loss observed here and the close biologic relationship between weight and glucose concentrations.

We observed a significant improvement in VO_2_max during the first three months of the trial. At 3 years the improvement in VO_2_max persisted only when expressed in absolute terms but not when expressed per unit body mass. This difference is likely to reflect the fact that when VO_2_max is expressed relative to weight, changes in VO_2_max are influenced by changes in body mass. This is partly because muscle is a highly oxidative tissue type, whereas adipose tissue is not. Thus changes in weight and in body composition impact aerobic capacity differently depending on whether the trait is expressed relative to weight or in absolute terms.

Reductions in waist circumference were observed in the absence of reductions in weight, which is consistent with results from other exercise studies [44]. Moreover, we observed no discernable impact of the intervention on lipid levels, which is consistent with the findings of other studies [9], [11], [17], [44]–[45]. However, in this study, lipid-lowering treatment was common and baseline lipid levels were consequently close to target levels, which may have masked any subtle changes in lipids resulting from the intervention.

We calculated the power available in our study to detect the same level of change in fasting glucose and HbA1c that was observed one-year post-randomisation in the DPP. In our study, the power to detect these changes was approximated 95% for changes in fasting glucose concentrations and 67% for changes in HbA1c at p<0.05. However, although this study was well powered to detect a clinically and statistically significant change in body weight, the magnitude of this change was less than the average amount of weight lost in the DPP. One would anticipate, therefore, that in the present study any effect of lifestyle intervention on fasting glucose levels or HbA1c would also be less, which likely explains the lack of effect on these traits observed here.

### Generalizability

One of the most important findings of the present study is that a program of lifestyle modification, using the core features of the DPP and Finnish DPS, can reduce cardiovascular risk factor levels in at-risk adults, even when applied in the conventional primary care setting, without additional resources. However, it is worth highlighting that although our cohort is likely similar in demographic characteristics to the cohorts enrolled in the Finnish DPS, which yielded comparable results to the DPP, the population in the northern part of Sweden is culturally and ethnically different from the cohorts enrolled into the DPP. It is also important to highlight that participants in the present study were invited by health care providers from their own health care centres to participate in this study, which may have facilitated recruitment rates. For example, more than half of those eligible to participate in our study were eventually randomised to treatment. By contrast, in a recent report from the same region of Sweden in which advertisements were used to recruit participants for a similar lifestyle intervention program, only 16 % of eligible individuals agreed to participate [46]. Thus, our strategy of recruiting through the established health care infrastructure may be fundamental to the success of our project and others that may follow.

It is also important to bear in mind that, as in all other lifestyle intervention trials, neither the participants nor the study staffs in this study were blinded to the allocation of treatment. Thus it is possible that placebo effects explain some of the beneficial changes in health associated with the lifestyle intervention. On the other hand, providing participants in the standard care arm of the trial with information about lifestyle and health, activity logs, and regular follow-up exams may have diluted differences between groups.

We present data analysed on an ITT basis, which is a highly conservative method for assessing the impact of an intervention. We also used mixed-models incorporating all available data without imputation of missing data points. Both methods yielded similar results, highlighting the robustness of our findings.

At baseline we asked the participants “if the beneficial effects of physical activity and drug treatment were the same, which method would you prefer?” and “if both physical activity on prescription and drug treatment were free of charge, which would you prefer?” The vast majority of our participants responded that they would prefer the physical activity option and none opted for drug therapy. This preference for physical activity as a form of treatment may reflect the cultural emphasis on healthy living in the north of Sweden. However, in both groups there was an increased use of lipid and blood pressure lowering medication during the study, which may reflect current treatment recommendations that emphasise reaching specific target levels.

Although this intervention was not designed to specifically promote smoking cessation, tobacco habits and the effects of tobacco on health were discussed at the follow-up meetings. That broad focus on healthy living may explain why more smokers quit in the lifestyle than in the control group. In combination with the reductions in waist circumference and systolic blood pressure observed in this trial and increased exercise and overall physical activity levels, the reductions in tobacco usage are likely to yield continuing improvements in the health of our participants.

Our study addresses the important objective of translating the findings from extensively resourced trials, which focussed on highly selected populations and study settings, to a real-life setting where resources are limited and the population is more heterogeneous. However, an inherent limitation of studies that seek to do this is that they suffer from lower internal validity. In this respect, the Björknäs study was not blinded and the intervention was less tightly controlled and less well resourced than several previous studies. In this study as in the DPP, only half of all eligible subjects were randomized to treatment [47]. However, those who declined to participate did not differ from those who took part with respect to all key characteristics. Because attrition rates were modest and we used ITT analyses, our findings may be less prone to bias than some other studies. Nonetheless, as indicated by the wide confidence intervals for several traits, our study was underpowered to detect small improvements in the clinical traits examined. Thus, some of our findings may be prone to type 2 error.

### Overall evidence

In summary, a lifestyle intervention program applied in the primary care setting beneficially impacts cardiovascular risk profiles. These findings provide some of the first empirical evidence that affordable programs of lifestyle intervention, which can be widely prescribed effectively reduce risk factors for cardiovascular disease in initially high-risk individuals. Our study also shows that such interventions can be integrated within existing health care infrastructures, at least in the Swedish setting. Studies such as ours support a de-emphasis of the use of drug therapies in favour of safer, more holistic, and more effective treatments combining diet and exercise prescription.

## Supporting Information

Checklist S1 CONSORT Checklist
(0.04 MB DOC)

Protocol S1 Trial Protocol
(0.04 MB DOC)
