# Supplementary material for: A 3-Year Randomized Trial of Lifestyle Intervention for Cardiovascular Risk Reduction in the Primary Care Setting: The Swedish Björknäs Study
Source: PLoS One. 2009 Apr 14;4(4):e5195. doi: 10.1371/journal.pone.0005195 (PMC2664964; doi:10.1371/journal.pone.0005195)
Supplement: Protocol S1 — Trial Protocol (0.04 MB DOC) [file pone.0005195.s002.doc]

Lifestyle Intervention in Primary Health Care - the Björknäs Study

This study has been completed.

Sponsors and Collaborators: Umeå University

County Council of Norrbotten, Sweden

Information provided by:Umeå University

ClinicalTrials.gov Identifier:NCT00486941

Purpose

The purpose of this study is to determine whether a short group intervention

programe aiming at lifestyle changes at a local health centre can improve risk

factors for cardiovascular disease

Condition Intervention Phase

Hypertension

Type 2 Diabetes

Obesity

Dyslipidemia

Behavioral: Exercise and diet - based on DPS and DPP

Phase III

MedlinePlus related topics: Diabetes Exercise and Physical Fitness

High Blood Pressure Obesity

U.S. FDA Resources

Study Type: Interventional

Study Design: Prevention, Randomized, Open Label, Active Control,

Parallel Assignment, Efficacy Study

Official Title: A Randomized Trial of Lifestyle Intervention in Primary

Health Care for the Modification of Cardiovascular Risk Factors - the

Björknäs Study

Further study details as provided by Umeå University:

Primary Outcome Measures:

Changes in anthropometry (BMI, waist and hip cf) [ Time Frame: 3 years ]

Maximal oxygen uptake (VO2max) [ Time Frame: 3 years ]

Health-related quality of life (EQ 5D, SF-36) [ Time Frame: 3 years ]

Self-reported physical activity [ Time Frame: 3 years ]

Secondary Outcome Measures:

Blood pressure [ Time Frame: 3 years ]

Total cholesterol, HDL and triglycerides [ Time Frame: 3 years ]

Glucose tolerance (OGTT) [ Time Frame: 3 years ]

Enrollment: 151

Study Start Date: February 2003

Study Completion Date: March 2006

Detailed Description:

Successfully transferring the findings of expensive and tightly controlled

lifestyle intervention programmes to the primary care setting is necessary if

such knowledge is to be used for disease prevention at the population level.

Therefore, our objective was to evaluate the efficacy of a lifestyle

intervention programe in the primary health care setting, targeted at patients

with moderate- to high-risk of cardiovascular disease according to

cardiovascular risk factor levels, physical activity and quality of life

ratings.

Randomised controlled trial with follow-up at 3, 12, 24 and 36 months, carried

out in a primary health care centre in Northern Sweden. A total of 151

middle-aged men and women, with hypertension, dyslipidemia, type 2 diabetes or

obesity were enrolled. The subjects were randomised to an intervention (n = 75)

or control group (n = 76). 120 subjects completed the three-year follow-up. The

intervention was based on the protocols used in the Finnish Diabetes Prevention

study (DPS) and the U.S Diabetes Prevention Program

Eligibility

Ages Eligible for Study: 18 Years to 65 Years

Genders Eligible for Study: Both

Accepts Healthy Volunteers: No

Criteria

Inclusion Criteria:

Patients from one single health care centre with a diagnosis of:

Typ 2 diabetes,

Hypertension,

Obesity or

Dyslipidemia

Exclusion Criteria:

Coronary heart

Disease,

Stroke,

TIA,

BP >180/105,

Dementia; or

Severe psychiatric disease

Contacts and Locations

Please refer to this study by its ClinicalTrials.gov identifier: NCT00486941

Locations

Sweden

Björknäs Health Centre

Boden, Sweden, SE-96164

Sponsors and Collaborators

Umeå University

County Council of Norrbotten, Sweden

Investigators

Principal Investigator: Mats CE Eliasson, MD, PhD Umeå University,

Umeå, Sweden

More Information

Publications of Results:

Margareta Eriksson K, Westborg CJ, Eliasson MC. A randomized trial of

lifestyle intervention in primary healthcare for the modification of

cardiovascular risk factors. Scand J Public Health. 2006;34(5):453-61.

Study ID Numbers: EPN-Umea 02-512

First Received: June 14, 2007

Last Updated: July 18, 2007

ClinicalTrials.gov Identifier: NCT00486941

Health Authority: Sweden: Institutional Review Board

Keywords provided by Umeå University:

Primary Prevention

Health Promotion

Diet

Exercise

Risk Factors

Randomized Controlled Trial

Study placed in the following topic categories:

Obesity

Metabolic Diseases

Diabetes Mellitus

Vascular Diseases

Endocrine System Diseases

Overweight

Body Weight

Signs and Symptoms

Diabetes Mellitus, Type 2

Nutrition Disorders

Overnutrition

Endocrinopathy

Metabolic disorder

Glucose Metabolism Disorders

Dyslipidemias

Lipid Metabolism Disorders

Hypertension

Additional relevant MeSH terms:

Cardiovascular Diseases

ClinicalTrials.gov processed this record on July 28, 2008

U.S. National Library of Medicine, Contact Help Desk

U.S. National Institutes of Health, U.S. Department of Health & Human

Services,

USA.gov, Copyright, Privacy, Accessibility, Freedom of Information Act

Links to all studies - primarily for crawlers
